# Hsp60 exerts a tumor suppressor function by inducing cell differentiation and inhibiting invasion in hepatocellular carcinoma

**DOI:** 10.18632/oncotarget.12185

**Published:** 2016-09-22

**Authors:** Jing Zhang, Xingchun Zhou, Hulin Chang, Xiaojun Huang, Xu Guo, Xiaohong Du, Siyuan Tian, Lexiao Wang, Yinghua Lyv, Peng Yuan, Jinliang Xing

**Affiliations:** ^1^ State Key Laboratory of Cancer Biology and Experimental Teaching Center of Basic Medicine, Fourth Military Medical University, Xi'an, Shaanxi, China; ^2^ Department of Hepatobiliary Surgery, Shaanxi Provincial People's Hospital, Xi'an, Shaanxi, China; ^3^ Department of Pain Treatment, Tangdu Hospital, Fourth Military Medical University, Xi'an, Shaanxi, China

**Keywords:** Hsp60, hepatocellular carcinoma, differentiation, invasion, mitochondrial biogenesis

## Abstract

Heat shock protein 60 (Hsp60), a typical mitochondrial chaperone, is associated with progression of various cancers. However, its expression and significance in hepatocellular carcinoma (HCC) remain largely unclear. In the present study, the mRNA and protein expression of Hsp60 in HCC tissues were detected by quantitative RT-PCR (n=24), western blot (n=7), and immunohistochemical staining (n=295), respectively. The correlation between Hsp60 expression and clinicopathological characteristics of HCC patient was also analyzed. Meanwhile, the influence of Hsp60 on malignant phenotype of HCC cells was further investigated. We found that expression of Hsp60 was significantly downregulated in HCC tissues compared to peritumor tissues. Hsp60 expression was significantly correlated with serum alpha -foetoprotein (AFP) level and tumor differentiation grade. Moreover, high Hsp60 expression cancer/pericancer (C/P) ratio was associated with a better overall survival rate (*P*=0.035, n=295). The prognostic implication of Hsp60 in HCC was further confirmed in another cohort of 107 HCC patients (*P*=0.027). Up-regulation of Hsp60 remarkably induced the cell differentiation and inhibited the invasive potential of HCC *in vitro and in vivo*. Intriguingly, the down-regulation of Hsp60 significantly impaired mitochondrial biogenesis. Although more data are required to clarify the underling mechanism responsible for function of Hsp60, our results suggested that the effect of Hsp60 on differentiation and invasion of HCC cells might be associated with mitochondrial biogenesis. Collectively, our findings indicated that Hsp60 exerted a tumor suppressor function, and might serve as a potential therapeutic target in the treatment of HCC.

## INTRODUCTION

Hepatocellular carcinoma (HCC) is one of the most common malignancies in Asia and Africa, especially in China [1, 2]. Recently, the incidence of HCC has increased in economically developed regions, including Western European countries, and United States [3–5]. In recent years, an enormous amount of research effort has been dedicated to establish new therapeutic strategies [6–8]. However, in most cases, for patients with HCC, surgical resection and liver transplantation remain the only curative treatment options. Therefore, to further explore novel molecular mechanisms underlying HCC tumorigenesis is still crucial for the development of novel therapeutic approaches.

Heat shock proteins (Hsps) belong to a functional superfamily, which are highly conserved during evolution and play important roles in protein folding and translocation [9, 10]. The superfamily members have been classified into Hsp90, Hsp70, Hsp60, Hsp40 and small Hsps including Hsp27 according to their molecular weight [11]. Among family members, Hsp60 has been identified to be located in mitochondria. The important role of Hsp60 in mitochondrial biogenesis is attributed to its essential function for assembly of proteins [12]. Recently, accumulating evidences have showed that function of Hsps expanded to tumor progression [8, 13, 14]. In particular, the expression of Hsp60 in tumor tissues has been implicated to be associated with progression of various cancers [15–18]. Current information on expression of Hsp60 and its role in HCC is very limited and it is even less for the mechanism on the role of Hsp60 in tumor.

In the present study, we evaluated the expression of Hsp60 in primary HCC using quantitative real-time PCR (qRT-PCR), western blot, and immunohistochemistry (IHC). Additionally, we analyzed the relationship between Hsp60 expression and the clinicopathological features of HCC, as well as prognostic value of Hsp60 in HCC patients. The effect of Hsp60 on malignant phenotype of HCC and underlying mechanism was also investigated.

## RESULTS

### Hsp60 expression is significantly decreased in human HCC

**Figure 1:**
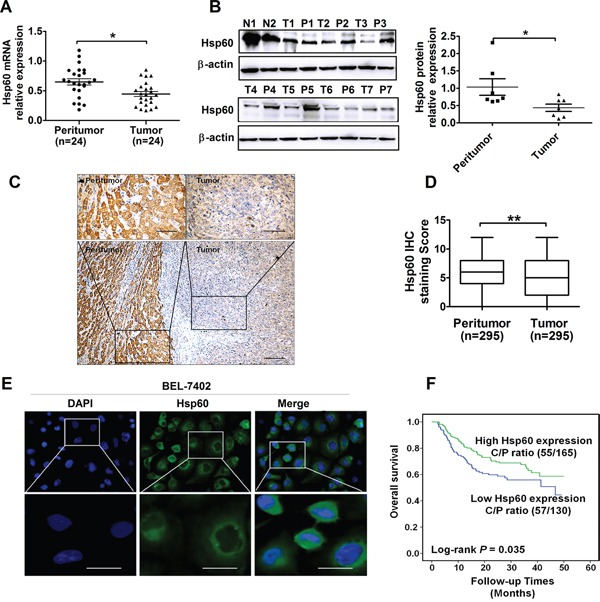
Hsp60 expression is down-regulated in HCC and predicts poor prognosis of patients **A-B.** qRT-PCR and western blot analyses for the mRNA and protein expression of Hsp60 in tumor and paired peritumor tissues from HCC patients. Quantitative analysis of protein levels was performed by measuring the ratio of Hsp60 to β-actin expression. Error bars indicate SD.*P<0.05. **C.** Representative immunohistochemical (IHC) staining images and **D.** IHC score of Hsp60 in paired HCC tissues (n=295; Scale bars, 100μm and 20μm, **P<0.01). **E.** Immunofluorescence analysis of the Hsp60 expression in BEL-7402 cell (Scale bars, 10μm). **F.** Kaplan-Meier curve of overall survival in 295 HCC patients based on Hsp60 expression ratio of cancer and pericancer tissues (C/P) (*P*=0.035). N: “normal human liver tissues” ; T: “tumor tissue” ; P: “peritumor tissues”.

Hsp60 mRNA expression was analyzed by qRT-PCR in 24 paired tumor and peritumor tissue samples from patients with HCC. We found that the mRNA expression level of Hsp60 in tumor tissue was much lower than that in peritumor tissue (Figure 1A). Consistent with the qRT-PCR result, western blot analysis indicated that the protein expression of Hsp60 was significantly decreased in HCC tumors (Figure 1B and 1C). Hsp60 expression was further evaluated by IHC in 295 pairs of tumor and peritumor tissues. The average IHC staining score of Hsp60 was 4.8 in HCC tissues, which was significantly lower than 6.8 of matched peritumor tissues (Figure 1D). All together, our data demonstrated that Hsp60 expression was reduced at both protein and mRNA levels in HCC patient-derived tumor tissues.

Based on the IHC staining results, Hsp60 was localized predominantly in the cytoplasm of tumor cells (Figure 1C and Figure S2), which was consistent with findings of other groups [6, 17]. Subcellular localization pattern of HSP60 in tumor cells was similar to that of peritumor cells. Moreover, the localization of Hsp60 was further confirmed primarily in the cytoplasm of human HCC cell BEL-7402 by immunofluorescence analysis (Figure 1E).

### Hsp60 down-regulation correlates with clinic pathological characteristics and survival of HCC patients

We further evaluated the clinical significance of Hsp60 expression in 295 HCC patients. The median score of Hsp60 immunostaining was 7 in peritumor tissues, whereas it decreased to 5 in tumor tissues. Patients were divided into low(<5) or high (≥5) Hsp60 expression groups according to the median IHC immunostaining score. The correlation between Hsp60 protein level and clinicopathologic features of HCC patients was analyzed. As listed in Table 1, 120 of 295 cases had low expression of Hsp60, while 175 of 295 cases had high expression of Hsp60. Moreover, we found that low Hsp60 expression was significantly correlated with high serum AFP level (*P*=0.020) and poor differentiation grade (*P*=0.004) of HCC patients. In contrast, Hsp60 expression level was not associated with age, gender, HBsAg status, tumor size, tumor number, TNM Classification of Malignant Tumor (TNM) stage, portal vein tumor thrombosis (PVTT) and relapse.

**Table 1:** The associations of Hsp60 expression with clinical characteristics of HCC patients

Variables	No. of cases	Hsp60 expression		P value (*χ^2^* test)
		Low	High	
**Total**	295	120	175	
**Age (years)**				0.620
<54	160	63	97	
≥54	135	57	78	
**Gender**				0.394
female	36	17	19	
male	259	103	156	
**HBsAg**				0.298
Negative	23	7	16	
Positive	272	113	159	
**AFP(ng/ml)**				**0.020**
<200	169	59	110	
≥200	126	61	65	
**Tumor size(cm)**				0.741
<5	122	51	71	
≥5	173	69	104	
**Tumor number**				0.596
Single	234	97	137	
Multiple	61	23	38	
**Differentiation grade**				**0.004**
I+II	78	21	57	
III+IV	217	99	118	
**TNM stage**				0.680
I+II	230	95	135	
III+IV	65	25	40	
**PVTT**				0.361
No	262	105	157	
Yes	33	15	18	
**Adjuvant TACE therapy**				0.855
No	218	88	130	
Yes	77	32	45	
**Death**				0.742
No	186	77	109	
Yes	109	43	66	
**Relapse**				0.450
No	108	47	61	
Yes	287	73	114	

AFP, alpha-fetoprotein; HBsAg, hepatitis B virus surface antigen; HCC, hepatocellular carcinoma; Hsp60, heat shock protein 60; PVTT, portal vein tumor thrombus; TACE, transcatheter arterial chemoembolization.

Considering that serum AFP level and differentiation grade are the main causative factor for HCC poor outcome, we further investigated whether Hsp60 expression could predict the prognosis of HCC patients. As shown in Figure 1F, Kaplan-Meier analysis indicated that HCC patients with low Hsp60 expression ratio of cancer/pericancer had a significantly poorer overall survival than those with high expression ratio of cancer/pericancer (C/P) (*P*=0.035). The prognostic implication of Hsp60 in HCC was further confirmed in another cohort of 107 HCC patients (Figure S3, *P*=0.027).

### Hsp60 induces the differentiation of HCC cells

**Figure 2:**
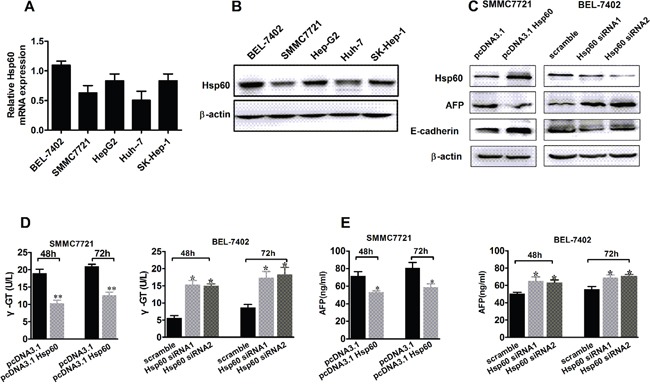
Hsp60 promotes the differentiation of HCC cells **A.** qRT-PCR and Western blot analysis **B.** of the Hsp60 expression in HCC cell lines. **C.** Western blot analysis for protein expression level of differentiation-related markers AFP and E-cadherin in BEL-7402 and SMMC7721 cells, which were transiently transfected with either Hsp60 siRNA or Hsp60 expression vector, respectively. **D.** γ-GT and **E.** AFP level in culture supernatant of BEL-7402 and SMMC7721 cells, which were treated as indicated. Data shown are the mean ± SD from three independent experiments, * *P*<0.05.

The expression of Hsp60 was assessed by qRT-PCR and confirmed by western blot in five HCC cell lines, including HepG2, BEL-7402, SMMC-7721, SK-Hep-1 and Huh7 (Figure 2A and 2B). Based on expression level of Hsp60, BEL-7402, SMMC-7721 and SK-Hep-1 cells were selected for the further experiment. To confirm the role of Hsp60 in differentiation of HCC cells, Hsp60 was overexpressed or silenced in either SMMC-7721 or BEL-7402 HCC cells, respectively. Then, the expression of γ-glutamyl-transferas (γ-GT), AFP, N-cadherin, Vimentin and E-cadherin was detected. γ-GT, AFP, N-cadherin and Vimentin are known to be specific for dedifferentiated cells with high grade of malignancy, while E-cadherin is recognized as one of the phenotypes specific for well-differentiated hepatocytes. As shown in Figure 2C, the differentiation marker E-cadherin significantly increased in SMMC-7721 cells with Hsp60 overexpression, whereas decreased in BEL-7402 cells with Hsp60 knockdown. In contrast, the level of dedifferentiation markers AFP, γ-GT, N-cadherin and Vimentin was conversely changed (Figure 2C and Figure S4). The expression level of E-cadherin was further analyzed in 20 paired HCC samples. As shown in Figure S5, the expression level of E-cadherin in the tumor tissues of HCC was much lower than that in peritumor tissues. In addition, ELISA assay also showed that γ-GT activity and AFP level were decreased in culture supernatant of SMMC-7721 cells with Hsp60 overexpression and increased in Hsp60-silenced BEL-7402 cells (Figure 2D and 2E). Collectively, our results indicated that overexpression of Hsp60 induced the differentiation of HCC cells.

### Hsp60 inhibits invasion and migration of HCC cells

**Figure 3:**
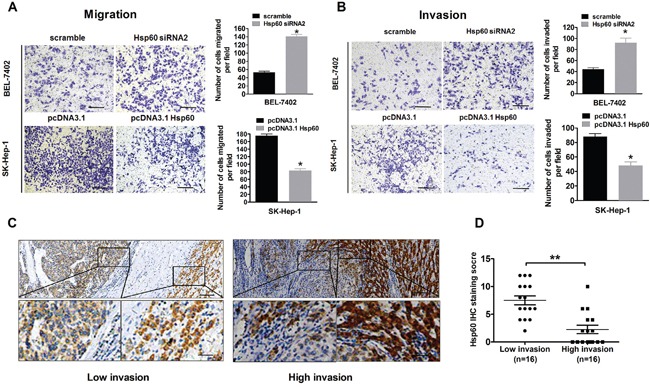
Ectopic expression of Hsp60 inhibits invasion and migration of HCC cells *invitro* **A-B.** Cell invasion and migration assays of BEL-7402 and SK-Hep-1 cells, which were transiently transfected with either Hsp60 siRNA or Hsp60 expression vector respectively. Values shown are expressed as the mean ± SD of three independent experiments, *P<0.05. Scale bars, 100 μm. **C.** Representative IHC staining images of Hsp60 in HCC tissues with high and low-invasive ability (Scale bars, 100μm and 20μm). **D.** Hsp60 IHC scores in HCC tissues with high and low-invasive ability.

During cancer progression, dedifferentiated tumor cells are often accompanied by the acquisition of cell motility and invasion. To explore the biological significance of Hsp60 in HCC invasion and metastasis, we knocked down Hsp60 in BEL-7402 and overexpressed it in SK-Hep-1 cells (Figure S1). The migration and invasion assay showed that the invaded and migrated cell number of BEL-7402 with Hsp60 knockdown was significantly higher than controls (*P*<0.05; Figure 3A and 3B). Conversely, overexpression of Hsp60 decreased the invasion and migration of SK-Hep-1 cells (*P*<0.05; Figure 3A and 3B). IHC analysis confirmed that HCC samples with high invasive potential had a significant decreased Hsp60 expression when compared with HCC samples with low invasive potential (Figure 3C and 3D). These findings suggested that Hsp60 was an important inhibitor for the migration and invasion of HCC cells.

**Figure 4:**
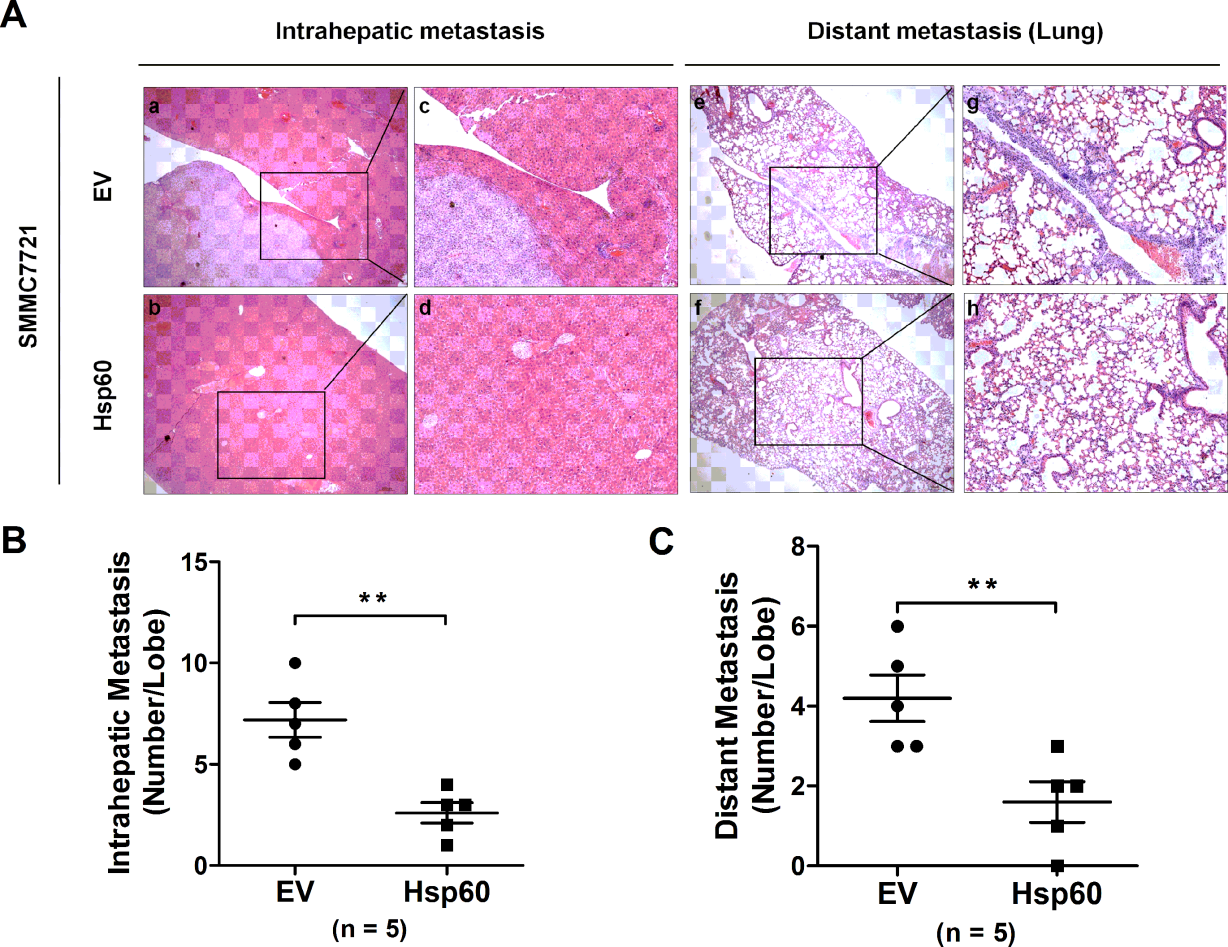
Hsp60 suppresses the metastatic potential of HCC cells *in vivo* **A.** Representative images of intrahepatic and distant metastatic nodules formed by SMMC-7721-Hsp60 cells or SMMC-7721-EV control are shown, along with the number of metastatic nodules in the livers or lungs (n=5 mice each group; original magnification 100×, c, d, g, and h, 400×). **B.** The numbers of metastatic nodules in each mouse liver (B) and lung **C.** were counted. (**P<0.01; EV: pcDNA3.1 empty vector; Hsp60: pcDNA3.1 Hsp60).

To further clarify the suppressive role of Hsp60 in HCC metastasis *in vivo*, the left hepatic lobes of nude mice were orthotopically inoculated with SMMC7721-Hsp60 and SMMC7721-EV cells. 8 weeks after the liver implantation, the mice were sacrificed. Histological examination of lung and liver tissues indicated that mice inoculated with SMMC7721-Hsp60 cells had significantly lower numbers of pulmonary and intrahepatic metastatic nodules than those inoculated with SMMC7721-EV cells (*P*<0.01; Figure S6 and Figure 4). These results showed that Hsp60 overexpression suppressed the metastatic potential of HCC *in vivo*.

### Hsp60 expression is associated with mitochondrial biogenesis

**Figure 5:**
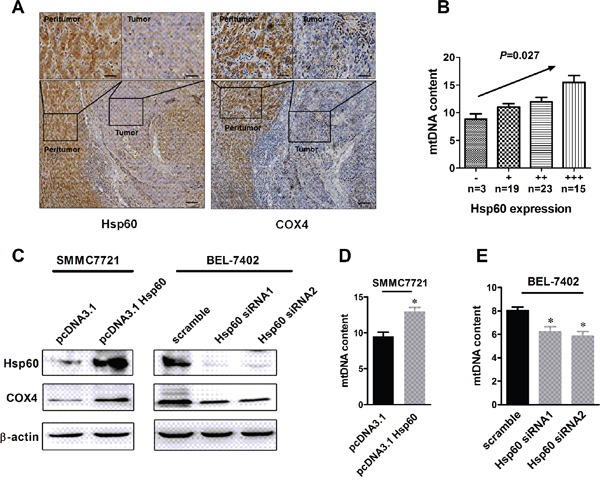
Hsp60 up-regulation is associated with increased mitochondrial biogenesis in HCC cells **A.** Representative immunohistochemical (IHC) staining images of Hsp60 and mitochondrial biogenesis marker COX4 (n=275; Scale bars, 100μm and 20 μm,). **B.** The correlation analysis between the protein expression levels of Hsp60 and mitochondrial DNA content in HCC tissues (n=60). **C.** Western blot analyses for protein levels of COX4 in HCC cells treated as indicated. **D**-**E.** Mitochondrial DNA content was analyzed in BEL-7402 and SMMC7721 cells treated as indicated.

Hsp60 is one of the most important mitochondrial protein which facilitates proper folding and assembly of newly synthesized protein during mitochondrial biogenesis. To determine whether function of Hsp60 in HCC was associated with mitochondria biogenesis, we measured the correlation between Hsp60 expression and the mitochondrial biogenesis, which was evaluated using mitochondrial biogenesis marker cytochrome c oxidase subunit (COX4) and mitochondrial DNA (mtDNA) content. Our data showed that Hsp60 expression was positively correlated with COX4 expresssion level (r=0.353, *P*< 0.001, n=275) and the mtDNA content (r=0.427, *P*=0.027, n=60) (Figure 5A, 5B and Table 2). These results were further confirmed in HCC cell lines, As shown in Figure 5C, 5D and 5E Hsp60 overexpression significantly increased COX4 expression and mtDNA content when compared with control cells, while COX4 expression and mtDNA content were decreased when Hsp60 was silenced by siRNA (Figure 5C, 5D and 5E). These results indicated that Hsp60 expression was associated with mitochondrial biogenesis.

**Table 2:** Correlation between Hsp60 and COX4 in HCC tissues

COX4 Expression	Hsp60 Expression				Total, N
	-	+	++	+++	
**-**	11	15	5	0	31
**+**	8	36	29	37	110
**++**	4	17	12	31	64
**+++**	3	15	11	41	70
**Total, N**	26	83	57	109	275

**Spearman correlation analysis, r = 0.353, *P*< 0.001.**

Since Hsp60 expression was positively correlated with the mtDNA content. Moreover, it has been proved that high Hsp60 expression cancer/pericancer (C/P) ratio was associated with a better overall survival rate. We further investigated whether mtDNA content was correlated with survival of HCC patients. As shown in Figure S7, patients with low mtDNA content had poorer OS than those with high mtDNA content (log-rank *P*=0.018 for OS).

### Hsp60 expression has not effect on proliferation and apoptosis of HCC cells

**Figure 6:**
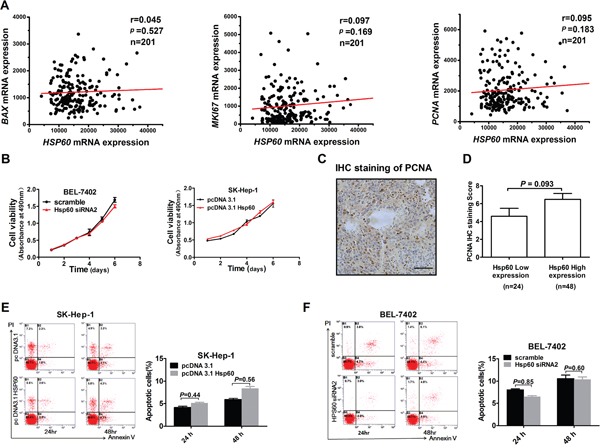
Hsp60 has no effect on proliferation and apoptosis of HCC cells **A.** Scatter plot analysis of correlation between mRNA expression levels of Hsp60 and BAX, KI67 and PCNA based on TCGA. Correlation coefficients (r) and levels of statistical significance (*P* value) were shown as indicated. **B.** MTS assay for the assessment of HCC proliferation ability. BEL-7402 and SK-Hep-1 cells were transiently transfected with Hsp60 siRNA and Hsp60 expression vector, respectively. Then cells were re-seeded for MTS cell viability assay 24 h after transfection. Absorbance at 490nm was measured at 1, 2, 3, 4, 5 and 6 days after reseeded. Values shown are the mean ± SD from at least three independent experiments. **C.** Representative IHC staining images of PCNA from HCC patient (Scale bars, 100μm). **D.** PCNA IHC score in HCC with low and high HSP60 expression. No statistically significant difference of PCNA expression level was observed between groups with high and low Hsp60 IHC score. **E, F.** Apoptosis analysis by flow cytometry in SK-Hep-1 and BEL-7402 cells treated as indicated by staining with Annexin V-FITC and propidium iodide (PI).

To explore whether Hsp60 was involved in HCC cell proliferation and apoptosis, we analyzed the correlation of mRNA expression between Hsp60 and proliferation or apoptosis-related genes, such as BCL2 associated X (BAX), KI67 and Proliferating cell nuclear antigen (PCNA) using TCGA database. As shown in Figure 6A, there was no correlation between Hsp60 and these genes. Subsequently, we assessed the effect of Hsp60 on proliferation ability of HCC cells. MTS assay indicated that no statistically significant difference in cell viability was found between BEL-7402 cells with Hsp60 knockdown and control cells. A similar result was also observed in SK-Hep-1 cells with Hsp60 overexpression (Figure 6B). These results were further confirmed by correlation analysis of IHC staining scores between Hsp60 and PCNA in HCC, indicating that there is no significant difference of PCNA expression between HCC samples with high Hsp60 expression and those with low Hsp60 expression (Figure 6C and 6D). Finally, we investigated the functional role of Hsp60 in HCC cell apoptosis. Our data showed that either increase of Hsp60 in SK-Hep-1 cells or decreased Hsp60 expression in BEL-7402 cells were well tolerated and did not cause apoptosis in HCC cells. (Figure 6E and 6F). Collectively, our results indicated that no apparent change in the proliferative or apoptotic ability of HCC cell occurred solely by alteration of Hsp60 expression.

## DISCUSSION

Here we demonstrated that Hsp60 expression was significantly decreased in HCC and associated with serum AFP level and tumor differentiation grade. High Hsp60 expression predicts favorable prognosis for HCC patients. Moreover, we found that Hsp60 induced the differentiation and inhibited invasion of HCC *in vitro and in vivo*. An insight into the mechanisms revealed that tumor suppressor function of Hsp60 might be associated with mitochondrial biogenesis. Our findings indicated that Hsp60 exerted a tumor suppressor function in HCC progression.

An aberrant Hsp60 expression has been found in various types of cancers [15-17, 19-24]. In addition, a few studies have addressed the Hsp60 expression in HCC. Using proteomics approach, Kuramitsu *et al* [25] have demonstrated that Hsp60 levels are increased in specimens of HCC patients with hepatitis C virus (HCV) infection, when compared with non-cancerous HCV-infected liver tissues. Moreover, another study has reported that no significant difference in Hsp60 expression was detected by IHC and dot immunoblotting between paired tumor and nontumoral specimens of 38 HCC patients with hepatitis B virus (HBV) infection [26]. Herein, we provided strong evidence that expression of Hsp60 was decreased in HCC tissues using a relatively large series of clinical tissue samples. The method and specific antibody used for assessing Hsp60 expression should be taken into consideration for the discrepancies among the different research groups. In addition, specific cohort of samples could be another reason for the disparate expression pattern of Hsp60. Compared to Kuramitsu's study, most of patients in our study were seropositive for hepatitis B surface antigen but none of patient had serum antibody against hepatitis C virus. Further investigations are required to clarify the influence of pathologic factors on the expression of Hsp60.

Although Hsp60 expression has been investigated in a large variety of tumors [17, 21, 27–30], so far systematic investigation of the prognostic significance of Hsp60 in HCC has not been reported, especially with relatively large series of tissue samples and sufficient follow-up data. In our study, the expression of Hsp60 was detected in 295 HCC patients based on IHC staining with 5 year's follow-up to verify its clinical relevance. we found that Hsp60 expression was significantly associated with differentiation grade of HCC tissues and serum AFP levels. Moreover, low level of Hsp60 predicted poor overall survival of HCC patients. To our knowledge, this is the first study to report the correlation between the expression of Hsp60 and prognostic factors of HCC in a large cohort of patients. Our results indicated that down-regulated Hsp60 expression contributed to the progression of HCC.

A series of reports have indicated that Hsp60 plays a critical role in the metastasis of various cancers [16, 30, 31]. In addition, data from other group have indicated that Hsp60 could help to distinguish dysplastic from normal tissue in tubular adenomas of the large bowel and stimulate vascular invasion, as well as enhanced lymph node metastasis in colorectal cancer [27, 29, 32, 33]. Consistent with these results, we found that ectopic expression of Hsp60 promoted the differentiation, whereas suppressed the invasive potential of HCC *in vitro* and inhibited both intrahepatic and lung metastasis *in vivo*. Moreover, Hsp60 knockdown by siRNA significantly enhanced HCC cell migration and invasion. These results were further confirmed by analyzing the correlation between Hsp60 expression and invasiveness in HCC tissues. As we know, the metastatic cell must exhibit phenotype of invading the surrounding normal tissue. Our IHC data showed that high expression of Hsp60 in tumor tissues was commonly accompanied by a less aggressive malignant phenotype, which typically exhibited well-defined normal-tumor boundaries. Except for the effect of Hsp60 on metastasis of HCC cells, the influence of Hsp60 on the differentiation of HCC cells was also explored. Our results indicated that ectopic expression of Hsp60 induced differentiation of HCC cells, which was consistent with correlation between expression Hsp60 and differentiation grade in HCC tissues. Tumor dedifferentiation is a well-known phenomenon, which has long been proposed to be involved in tumor invasion [34]. It has been reported that the process of dedifferentiation was an essential step in acquiring cancer stem cell phenotypes characterized by enhanced invasive properties [35]. Noteworthy, overexpression of Hsp60 in HCC cells did not affect cell proliferation and apoptosis. Based on these findings, we proposed that Hsp60 functioned as a tumor suppressor during the progression of HCC by inhibiting invasion and inducing differentiation of HCC cells.

Hsp60 functions as a molecular chaperone to assist the folding and assembly of mitochondrial proteins [36], by which it contributes to mitochondrial biogenesis. The important role of Hsp60 in mitochondrial biogenesis was initially identified by the analysis of temperature sensitive mutants [12]. Furthermore, the affiliation of Hsp60 with mtHSP70 emphasizes its critical role in mitochondrial biogenesis [37]. Consistent with these findings, we found that expression of Hsp60 was positively correlated with that of mitochondrial biogenesis marker COX4 as well as mtDNA copy number in HCC tissues and ectopic expression of Hsp60 enhanced expression of COX4 and increased mtDNA copy number in HCC cells. Accumulating evidence has indicated that reduced expression of mitochondrial regulator gene, such as α subunit of peroxisome proliferators-activated receptor-γ coactivator- 1(PGC1α), was associated with invasion of tumor, suggesting a role for abnormal mitochondrial biogenesis in tumor metastasis [38–41]. Decreased expression of PGC1αis required for invasive and metastatic cancer cells to acquire migratory phenotype. Moreover, a very recent study suggested that metastasis suppressor Kisspeptin-1 (KISS1) could reverse the Warburg effect by enhancing mitochondrial biogenesis through regulating PGC1α [42]. In line with these findings, our study demonstrated that higher mtDNA content was associated with poor prognosis of HCC patients. Therefore, we speculated that Hsp60 might suppress metastasis by enhancing mitochondrial biogenesis in HCC cells. Although more data are required to demonstrate the connection between tumor suppressor role of Hsp60 and mitochondrial biogenesis, our results indicated that invasion properties of HCC cells might be associated with mitochondrial biogenesis.

In conclusion, our findings demonstrate that Hsp60 may play an important role in invasion, differentiation and prognosis of HCC. Further investigation indicated that Hsp60 might exert a tumor suppressor function in hepatocellular carcinoma via promoting mitochondrial biogenesis. Our data suggested that Hsp60 could work as a promising target for prognostic prediction in HCC. However, much more experiments needs to be performed to put forward the diagnostic or therapeutic use of Hsp60.

## MATERIALS AND METHODS

### Patients and follow-up

Tumor and peritumor specimens of 295 HCC patients undergoing curative resection were collected from February 2009 to December 2012 in the Eastern Hepatobiliary Surgery Hospital, Shanghai, China. Another 107 pairs of tumor and peritumor specimens of HCC patients were collected from January 2007 to January 2009 in Xijing Hospital, Fourth Military Medical University in Xi'an, China. Patients who met the following criteria of eligibility were included in our study: (1) primary HCC diagnosed by histopathological examination; (2) treatment with radical resection; (3) availability of complete follow-up data; (4) no prior anticancer treatment; such as chemotherapy and radiotherapy; (5) availability of suitable formalin fixed, paraffin-embedded tissues and frozen tissues; and (6) no history of familial malignancy or other synchronous malignancy. The histopathological type and grade were determined using the criteria of the World Health Organization (WHO) classification [43]. Liver function was assessed by Child-Pugh classification. Tumor staging was defined according to the seventh-edition of Tumor-Node-Metastasis (TNM) Classification of the Union for International Cancer Control (UICC) and American Joint Committee on Cancer (AJCC) [44]. The study was approved by the Ethical Committee of Second Military Medical University, and informed consent was obtained from each patient.

Clinicopathological data of all patients, including gender, age, background liver pathology, serum AFP level, ALT, liver function, tumor size, number, vascular invasion, capsule, differentiation and stage, were collected from medical records. The patients were followed up by telephone or outpatient review. The clinical data included symptoms, serum AFP levels, imaging of abdominal ultrasound and chest radiograph. The deadline of follow-up was August 1, 2013. Overall survival was defined as the interval between surgery and either death or the last observation taken. The characteristics of these patients were listed in Table 1.

### HCC tissues and cell lines

HCC and paired peritumor tissues were collected from HCC patients. After resection, the fresh tissues were immediately frozen in liquid nitrogen and stored at -80°C. Both the HCC and peritumor tissues were verified by histopathological examination. Human HCC cell lines HepG2, BEL-7402, SMMC-7721, SK-Hep-1 and Huh7 were purchased from the Shanghai Institute of Cell Biology. Cells were cultured in RPMI 1640 or DMEM supplemented with 10% fetal bovine serum and maintained in 5% CO_2_ at 37°C.

### RNA interference

Cells were transfected with 20 nM siRNA using Lipofectamine RNAiMAX transfection Reagent (Invitrogen, #13778-075) according to the manufacture's protocol. At 48h after transfection, the cells were harvested and subjected to further analyses. All siRNAs were synthesized by GenePharma (Shanghai, China). The sequences of Hsp60-targeting siRNAs are as follows: Hsp60 siRNA1 5′-CCGAGCCUUAAUGCUUCAATT-3′, 5′-UUGAAGCAUUAAGGCUCGGTT-3′; Hsp60 siRNA2 5′-GUUGUGAGAACUGCUUUAUTT-3′, 5′- AUAAAGCAGUU CUCACAACTT -3′. A non-targeting siRNA (Scramble) was used as a control: 5′-UUCUCCGAACGUGUCACGUTT-3′, 5′-ACGUGACACGUUCGGAGAATT-3′.

### Plasmid construction and transfection

Open reading frame of human Hsp60 gene (NM_199440.1) was amplified by PCR using cDNA from BEL-7402 cells as template. The primer sequences used were 5′-GGGGAAGCTTGCCACCATGCTTCGGTTACC-3′ and 5′-GGGGATCCTTAGA ACATGCCACCTCCCATAC-3′. The PCR was conducted as follows: denaturation at 95 °C for 5 min, followed by 30 cycles of 95 °C for 30 s, annealing at 56°C for 30s and extension at 72 °C for 2min. After that, the PCR fragment was purified and ligated into Hind III and Bam H1-digested pcDNA3.1(+) vector to produce pcDNA3.1-Hsp60. After sequence confirmation, pcDNA3.1-Hsp60 and pcDNA3.1 control vector were transiently transfected into cells using lipofectamine 2000 (Invitrogen,#11668-027) according to the manufacturer's instructions. At indicated time points after transfection, the cells were harvested and subjected to further analyses.

To construct stable cell lines, SMMC7721 cells were transfected with recombinant plasmid pcDNA3.1-Hsp60 by Lipofectamine 2000 (Invitrogen) according to the manufacturer's instructions. The cells transfected with empty vector were used as controls. Stable cell lines were selected with G418 (800μg/ml; Sigma) and individual clones were isolated and maintained in medium containing G418 (400μg/ml). The stable clones were named as SMMC7721-Hsp60 (transfected with pcDNA3.1-Hsp60) and SMMC7721-EV (transfected with pcDNA3.1 empty vector).

### *In vitro* migration and invasion assays

Cell invasion and migration ability was assessed by transwell assays (Millipore, Billerica, MA). In brief, HCC cells were transfected with siRNA targeting Hsp60 or Hsp60 expression plasmid. The cells transfected with scramble siRNA or empty vector were used as control. At 24 h after transfection, the cells were suspended in culture medium at a density of 2 × 10^5^ cell/mL. Then 200μL cell suspensions were seeded into the upper chambers of the transwell, in which the porous membrane was either coated with Matrigel (BD Bioscience, # 356234) for the invasion assays, or left uncoated for the migration assays. Culture medium with 10% serum was added to the bottom chamber as a chemoattractant. After migration or invasion for 24 h, the cells that had penetrated the filters were fixed in 95% alcohol, and stained with crystal violet. Stained cells were counted for five fields per filter under a phase contrast light microscope (Olympus) and photographed at ×10 magnification. All experiments were performed in triplicate.

### MTS assay

For MTS assay, the HCC cells were transfected with either siRNA targeting Hsp60 or Hsp60 expression plasmid for 24 h. Transfected cells were collected and reseeded at a density of 3000 cells/well in 96-well culture plate. Cell viability was determined by the MTS assay (Promega Corp., #G5421) according to the manufacturer's instructions. The microplates were read in a spectrophotometer at a wave length of 490 nm. Each treated or control group contained 6 parallel wells and all experiments were performed in triplicate.

### Apoptosis assay

Cell apoptosis was determined by Annexin V-FITC Apoptosis Detection Kit (BestBio,#401001) according to the manufacturers' instructions. Briefly, HCC cells seeded in 6-well plates were collected and resuspended with 500 μL binding buffer at a concentration of 10^6^ cells/mL. After adding 5μL Annexin V-FITC and 5μL PI, cells were mixed and incubated at room temperature in the dark for 15 min. The samples were analyzed with a flow cytometry (Beckman). The CellQuest software was used to determine the percentage of apoptotic cells. Each treated or control group contained 3 parallel wells and all experiments were performed in triplicate.

### Western blot analysis

The total protein of cell or tissue lysate was isolated as previously described [45]. Cells were harvested in radioimmunoprecipitation (RIPA) assay buffer(Santa Cruz Biotechnology,#sc-24948). Protein content was quantified using the Bradford assay with BSA as standard (Bio-Rad), and 30μg of total protein lysate were separated on SDS-PAGE and transferred to polyvinylidene difluoride (PVDF) membrane. After blocking (5% non-fat milk in PBS, 1 h at RT), membranes were incubated at 4°C overnight with the following primary antibodies: Hsp60 antibody (Affinity, #AF0184,1:1000), β-actin monoclonal antibody (Kangchen, #KC-5A08,1:5000), AFP antibody(Abcam, #3980, 1:1000), E-cadherin antibody (proteintech,# 20874-1- -AP,1:1000), Vimentin Antibody (proteintech, #10366-1-AP,1:1000), N-Cadherin antibody (Abcam, #ab18203) and COX4 antibody (Abgent, #ALS12730,1:1000). After three washes with PBS, membranes were incubated with peroxidase-labelled secondary antibodies (Merck Millipore, 1:5000) at 37° C for 1 h. Immunoblots were developed using the enhanced chemiluminescence reagent (Pierce, #32209) .

### RNA extraction and quantitative real-time polymerase chain reaction(qRT-PCR)

Total RNA was isolated from HCC tissues and cells using TRIzol® (Invitrogen), treated with DNAse (Promega) and used for reverse transcription using SuperScript® II Reverse Transcriptase (Invitrogen, #18064-014) according to manufacturer's protocol. The resulting cDNA was used for qRT-PCR analysis to evaluate the relative expression levels of Hsp60 and β-actin using the following primers: 5′-AAGCTCTAAGTACACTCGTCTTGAATAGG- 3′and 5′-GCACCACCAGTAGCAATAGCCATAT- 3′ for Hsp60; 5′-TGACCCAGATCATGTTTGAG- 3′and CGTACAGGGATAGCACAG- 3′ for β-actin. The qRT-PCR reactions and analyses were carried out in 7500 Sequence Detection System (Applied Biosystems). cDNA was amplified using a SYBR Green PCR Kit (Takara, #4309155). Each 25μL of reaction volume contained 2μL of cDNA that was synthesized as mentioned, 12.5μL of SYBR Green mix, and 10μM of each pair of oligonucleotide primers described above. The cycling parameters began with an initial denaturation step at 95°C for 5 min; followed by 40 cycles of 95°C for 15 s, 60°C for 30 s and 72°C for 15 s. Relative mRNA expression levels were calculated by the 2^−ΔCt^ method based on the threshold cycle (Ct) values and were normalized to the internal control of β-actin.

### Immunohistochemical staining

Paraffin-embedded tissues were sectioned and immersed in boiled citrate-disodium hydrogen phosphate buffer (pH=6.0) with high pressure for 5 minutes for antigen retrieval. The sections was incubated with polyclonal rabbit anti-human Hsp60 (Affinity, #AF0184,1:100), anti-COX4 (Abgent,#ALS12730,1:100), E-cadherin antibody (proteintech, # 20874-1- -AP,1:100), or anti-PCNA (Abcam, #18197, 1:200) overnight at 4°C. Sections were incubated in biotinylated secondary antibody and streptavidin peroxidase. For color development, fresh 3,3′-diaminobenzidine (DAB) solution was added to tissue slices, followed by counterstaining with hematoxylin. IHC staining was scored by two independent pathologists (Xingchun Zhou and Xiaojun Huang) who are blinded to the clinical characteristics of the patients. In brief, the percentage of positive stained cells were scored as 0 (0-9%), 1 (10%-25%), 2 (26%-50%), 3 (51%-75%) or 4 (76%-100%), and the intensity as 0 (no staining), 1 (weak staining), 2 (moderate staining) or 3 (dark staining). The total score was calculated as the product of intensity and extent, ranging from 0 to 12. A total score of < 1, ≥ 1 to < 4, ≥ 4 to <8, and ≥ 8 was defined as being negative (-), weak positive (+), moderate positive (++), and strong positive (+++), respectively.

### Immunofluorescence Staining

For immunofluorescence analysis, cells were seeded on coverslips in a 24-well plate (1×10^5^ cells/well) and allowed to adhere overnight. Cells were then washed, fixed, and blocked in 1% bovine serum albumin for 1 hour. The slides were incubated with polyclonal rabbit anti-human Hsp60 antibody overnight at 4°C. After three washes with PBS, slides were incubated in fluorescein isothiocyanate (FITC)-conjugated goat anti-rabbit antibody (Abcam, #ab7050) for 1 h and then stained with 4′,6-diamidino-2-phenylindole (DAPI) for 10 min. The cells were then analyzed by fluorescence microscopy.

### Measurements of γ-GT and AFP

HCC cells were grown at a density of 1×10^6^ cells/ml and allowed to grow for 24 h before transfection with the Hsp60 siRNA or the expression plasmid. The cells treated with pcDNA3.1 vector or scramble siRNA were served as controls. At 48h after transfection, culture medium was harvested for measuring the contents of γ-GT and AFP. The activities of γ-GT were determined using commercially available kits according to the manufacturer's instructions (Jian Cheng, China, #C017-2). The concentrations of AFP were measured using an ELISA kit according to the manufacturer's instructions (CUSABIO, # CSB-E0477).

### Determination of mtDNA content by qRT- PCR

Relative mtDNA content in HCC cells was measured by a qRT-PCR-based method as previously described [46], with the same primers that were used for the mitochondrial ND1 gene (ND-R and ND-F) and the single-copy nuclear gene human globulin (HGB-1and HGB-2).

### *In vivo* metastasis assays

6-week-old male nude mice (BALB/c) were randomly divided into groups (5 mice/group). For *in vivo* metastasis assay, nude mice were anesthetized with 2.5% sodium pentobarbital (40mg/Kg; Sigma-Aldrich). Then, 2×10^6^ SMMC7721-Hsp60 (transfected with pcDNA3.1-Hsp60), and SMMC7721-EV (transfected with pcDNA3.1 empty vector) cells were suspended in 50mL PBS and orthotopically inoculated into the left hepatic lobe of nude mice. After 8 weeks, the mice were sacrificed and the number of intrahepatic metastatic foci in hepatic lobes other than the injected lobe and the number of distal lung metastasis foci was counted by double-blindly microscopical H.E staining evaluation as previously described [7]. Housing and all other procedures were performed according to protocols approved by the Animal Experimentation Ethics Committee of the Fourth Military Medical University. All animals received human care and study protocols comply with the institution's guidelines.

### Statistical analysis

SPSS 17.0 software (SPSS, Chicago, Illinois, USA) was used for all statistical analyses. Differences between the mean of two groups were compared using Student's *t* test. One-way ANOVA followed by a Newman Keuls' multiple comparison test was used to compare γ-GT and AFP level between control group and experimental group. The expression of Hsp60 in cancer tissues was divided into high or low level by the median value of immunostaining score for further analysis. Chi-squared test was used to analyze the associations between Hsp60 expression and various clinicopathological parameters. The Kaplan-Meier curve was used to estimate the overall survival (OS). Group differences were analyzed with the log-rank test. Correlation between measured variables was tested by Spearman's rank correlation analysis. All statistical tests were two-sided. *P*<0.05 was considered to be statistically significant.

## SUPPLEMENTARY MATERIALS FIGURES


