# Supplementary material for: Hsp60 exerts a tumor suppressor function by inducing cell differentiation and inhibiting invasion in hepatocellular carcinoma
Source: Oncotarget. 2016 Sep 22;7(42):68976–89. doi: 10.18632/oncotarget.12185 (PMC5356605; doi:10.18632/oncotarget.12185)
Supplement: Supplementary file 1 [file oncotarget-07-68976-s001.pdf]

Hsp60 exerts a tumor suppressor function by inducing cell differentiation and inhibiting invasion in hepatocellular carcinoma

SUPPLEMENTARY FIGURES

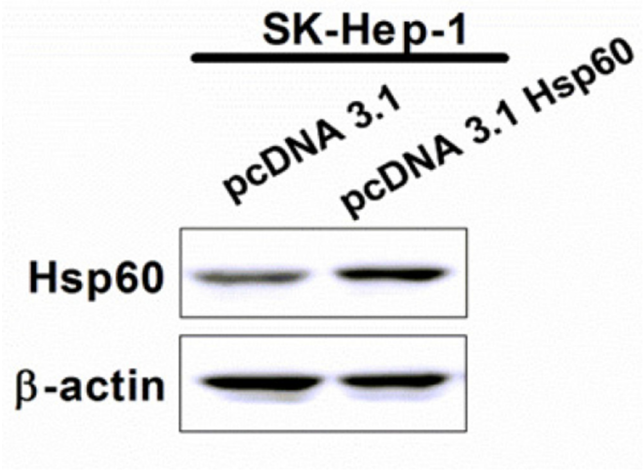

Supplementary Figure S1: Western blot analysis for protein expression of Hsp60 in HCC cells. Hsp60 protein expression was analyzed in SK-Hep-1 cells transiently transfected with an expression vector pcDNA3.1 Hsp60 or pcDNA3.1 empty vector.

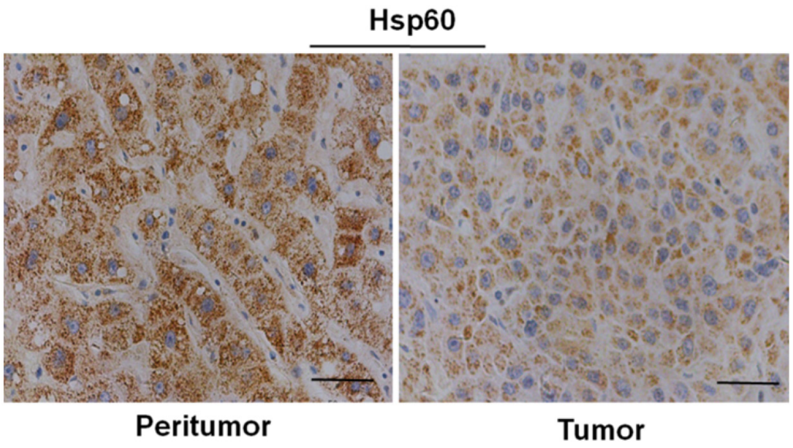

Supplementary Figure S2: Representative Hsp60 immunohistochemical (IHC) staining images of HCC and peritumor tissues. The IHC staining score of these sections were calculated according description in "MATERIALS AND METHODS". (n=295; Scale bars, 20μm).

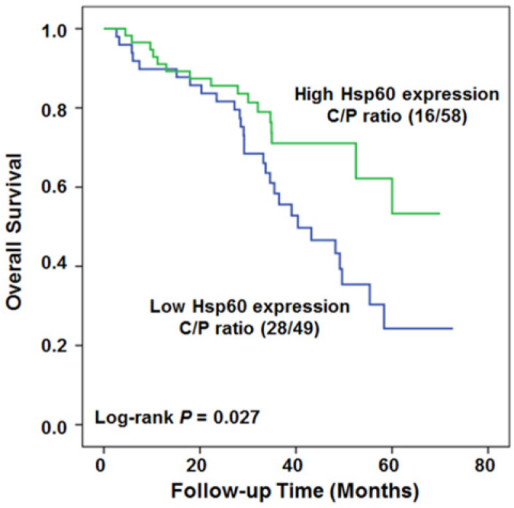

Supplementary Figure S3: Kaplan-Meier curve of overall survival (OS) in HCC patients based on Hsp60 expression ratio of cancer and pericancer tissues (C/P) (n=107; *P*=0.027).

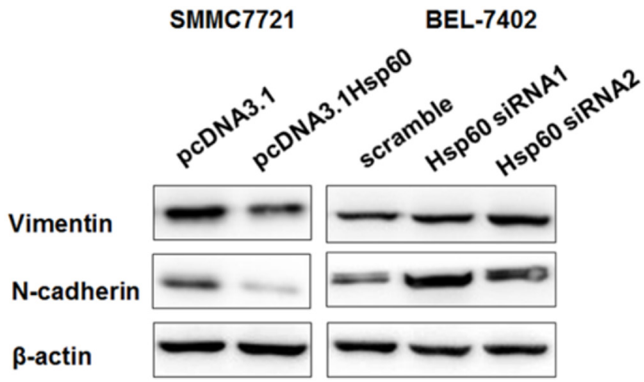

Supplementary Figure S4: Western blot analysis of differentiation-related markers Vimentin and N-cadherin in BEL-7402 and SMMC7721 cells. BEL-7402 and SMMC7721 cells were transiently transfected with either Hsp60 siRNA or Hsp60 expression vector, respectively.

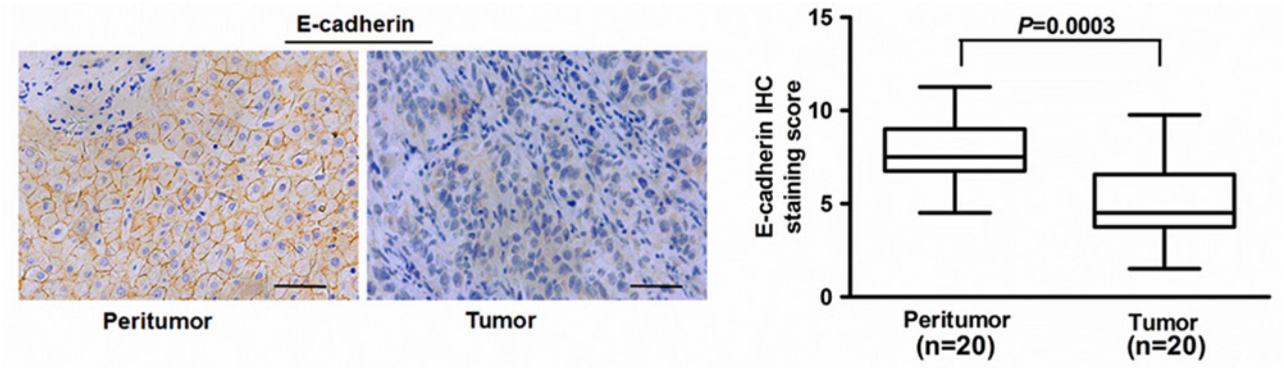

Supplementary Figure S5: Representative immunohistochemical (IHC) staining images and IHC score of E-cadherin in paired HCC tissues(n=20; Scale bars, 20μm).

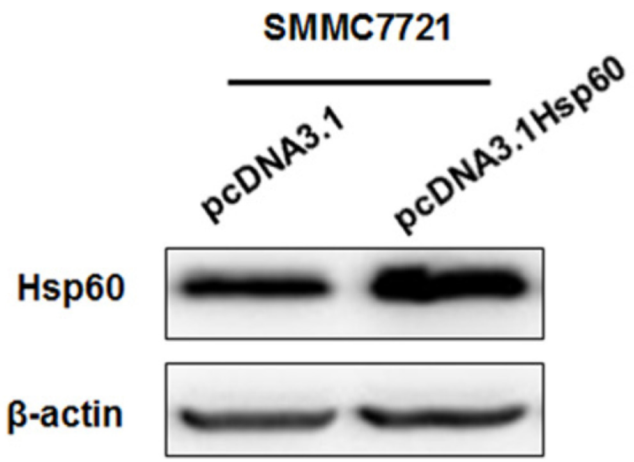

Supplementary Figure S6: Western blot analysis for protein expression of Hsp60 in HCC cells. Hsp60 protein expression was analyzed in SMMZ7721 cells stable transfected with expression vector pcDNA3.1 Hsp60 or pcDNA3.1 empty vector.

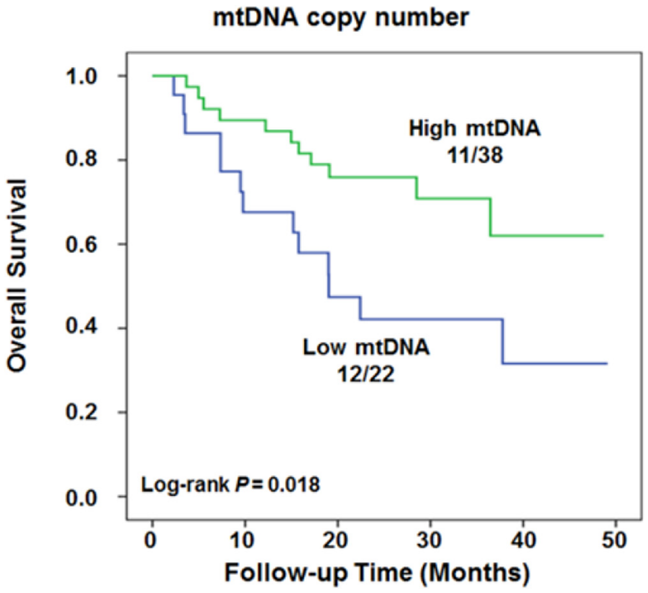

Supplementary Figure S7: Kaplan–Meier curves of overall survival (OS) in 60 HCC patients with different mtDNA content (*P*=0.018).
